# Isolation of *Lodderomyces elongisporus* from the Catheter Tip of a Fungemia Patient in the Middle East

**DOI:** 10.1155/2013/560406

**Published:** 2013-04-03

**Authors:** Suhail Ahmad, Zia U. Khan, Molly Johny, Najat M. Ashour, Wehad H. Al-Tourah, Leena Joseph, Rachel Chandy

**Affiliations:** ^1^Department of Microbiology, Faculty of Medicine, Health Sciences Center, Kuwait University, P.O. Box 24923, 13110 Safat, Kuwait; ^2^Department of Microbiology, Al-Amiri Hospital, Ministry of Health, P.O. Box 4077, Safat 13041, Kuwait; ^3^Department of Medicine, Al-Amiri Hospital, Ministry of Health, P.O. Box 4077, Safat 13041, Kuwait

## Abstract

*Lodderomyces elongisporus* is phenotypically closely related to *Candida parapsilosis* and has recently been identified as an infrequent cause of bloodstream infections in patients from Asia and Mexico. We report here the isolation of *Lodderomyces elongisporus* from the catheter of a suspected case of fungemia. The identity of the isolate was confirmed by phenotypic characteristics and ribosomal DNA sequencing.

## 1. Introduction

The ascomycetous yeast *Lodderomyces elongisporus *has long been considered as a teleomorph of *C. parapsilosis *due to various phenotypic similarities that it shares with *Candida parapsilosis*. Early molecular studies based on comparison of nDNA relatedness, however, suggested that they represent two separate species [1]. Subsequent taxonomic studies also suggested that *C. parapsilosis* form II (now redesignated as *Candida orthopsilosis*) is the anamorph of *L. elongisporus* [2]. This observation was also supported by comparative isozyme studies and nDNA complementarity data [3, 4]. Subsequently, 18S rRNA gene sequence analysis confirmed that *L. elongisporus* is a distinct species which is closely related (>97.5% sequence identity) to *C. parapsilosis* [5]. Further molecular studies based on multigene sequence analyses of several *Candida* species placed *L. elongisporus *in a clade that comprises *C. parapsilosis, C. albicans, *and* C. dubliniensis *[6]. It is the only species in this clade which forms ascospores. So far, only little information is available about the geographic distribution of this species. In a survey of 542 clinical *C. parapsilosis* isolates obtained from 25 countries, 10 strains were identified as *L. elongisporus*, 8 of which originated from a single hospital in Mexico and two each from China and Malaysia [7]. Interestingly, all 10 *L. elongisporus *isolates were recovered from blood samples, thus underscoring the role of this species in the etiology of candidemia. More recently another case of bloodstream infection from a patient in Malaysia and a case of endocarditis from Australia have been reported due to *L. elongisporus* [8, 9]. Here, we describe the isolation of *L. elongisporus* from the Middle East. The isolate was recovered from the tip of an intravascular catheter of a suspected case of fungemia.

## 2. Case Report

A 63-year-old Kuwaiti male was unconscious upon arrival in the hospital. On regaining consciousness, he appeared confused and disoriented. On clinical examination, he was hypotensive (90/60 mmHg) and hypothermic (32-33°C). There were no detectable abnormalities in cardiovascular system, gastrointestinal system, or respiratory system. Neurologically, he had slurred speech and diminished reflexes. There were no meningeal signs and fundoscopic examination was normal. He gave no history of smoking, alcoholism, or drug abuse. However, he had a history of schizophrenic-like condition and received treatment in a psychiatry hospital as outdoor or indoor patient. His chest X-ray was normal, but electrocardiogram revealed bradycardia. The CT scan of the brain showed lacunar infarcts in frontal lobes and right basal ganglia, and also ill-defined hypodensities in right parietal area suggesting evolving infarct. There was also evidence of mild encephalopathy by electroencephalogram. Complete blood counts as well as kidney and liver function parameters were within normal limits. Clinically, he was diagnosed to have cardiovascular attack and left hemiparesis with seizure. Besides administration of intravenous fluid, a nasogastric tube was fixed for nutritional purposes. During hospital stay, the patient became febrile (38.5°C) with signs of aspiration pneumonia. A blood specimen was collected from peripheral vein for aerobic and anaerobic culture and treatment with ceftriaxone and clindamycin was initiated. A second blood sample was collected two days later as the patient continued to remain febrile.

One set of the aerobic Bactec blood culture bottle (Beckton-Dickenson, Sparks, MD) from the first sample yielded a yeast growth after 89 h of incubation. Pending specific identification of the yeast (isolate Kw554-08), the patient's treatment was started with intravenous fluconazole (Diflucan) 400 mg/day along with removal of central venous catheter. The culture of central venous catheter tip removed from the patient also yielded yeast growth (isolate Kw553-08). While the bloodstream yeast isolate Kw554-08 remained unidentified by Vitek 2 yeast ID system (bioMérieux, Marcy l'Etoile, France), the catheter tip isolate Kw553-08 was identified as *C. parapsilosis*. Following 10 days of fluconazole therapy, the patient showed clinical improvement and became afebrile. The repeat blood cultures did not yield any bacterial or fungal growth. He was discharged in a stable condition with the advice to continue with physiotherapy.

## 3. Phenotypic Identification of Yeast Isolates

**Figure 1:**
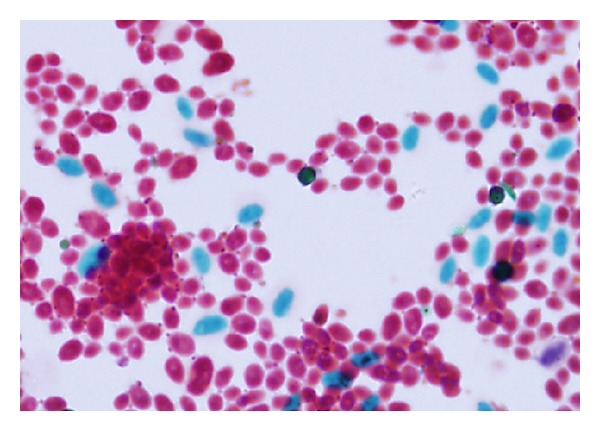
Ellipsoidal to elongate ascospores (green) of *L. elongisporus* produced on cornmeal agar and stained with Schaeffer-Fulton stain. Magnification, ×1000.

Both catheter tip and blood culture yeast isolates produced cream-colored colonies on Sabouraud dextrose agar. The catheter tip isolate Kw553-08 was identified as *C. parapsilosis *by Vitek2 yeast identification system (bioMérieux). However, it produced turquoise blue color colonies on CHROMagar Candida (Becton Dickinson), which were different in color from those produced by the typical isolates of *C. parapsilosis*. Thus, the identity of isolate Kw553-08 required further confirmation. The isolate was cultured on Corn Meal agar as well as on Malt extract agar (Becton Dickinson). After 10 days of incubation at 30°C, the isolate Kw553-08 formed long, ellipsoidal-shaped ascospores (Figure 1), thus suggesting its identity as *L. elongisporus.* Since the API 20C and ID 32C (bioMérieux) yeast identification systems do not include carbon assimilation profile for *L. elongisporus, *it was erroneously identified as* C. parapsilosis.*

**Figure 2:**
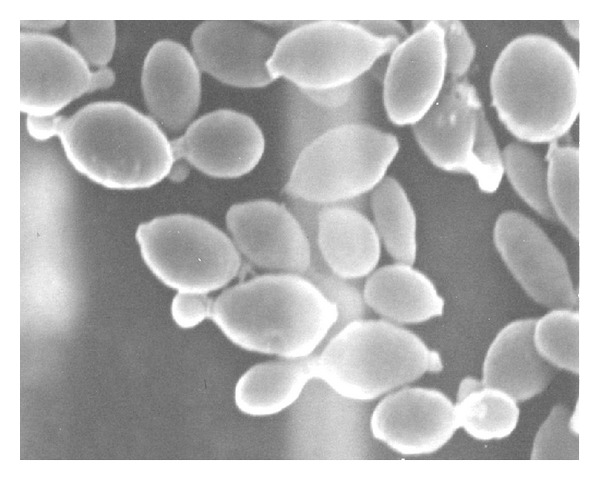
Scanning electron micrograph of *S. elviae* grown on malt extract broth showing separation of yeast cells by septa formation.

The blood culture isolate Kw554-08 showed spherical to oval yeasts (4–6 × 4–9 *μ*m) that formed conidia on short stalks, which eventually separated by septa formation (Figure 2). On Sabouraud dextrose agar, the isolate grew well at 30°C and 37°C and also produced detectable growth at 40°C. On cornmeal agar at 30°C, no pseudohyphae or hyphae were observed. The carbon assimilation profile obtained with the API 20C yeast identification system yielded a code of 6332022, whereas the ID 32C system yielded a code of 44221050 after 48 h of incubation at 30°C. However, neither of these codes could identify the isolate Kw554-08 to species level. Thus, the isolate remained unidentified by API 20C and ID 32C as well as by Vitek 2 yeast identification systems. Since the isolate Kw554-08 was positive for urease activity, it was considered to be a basidiomycetous yeast species. With a view to obtaining unequivocal identification, both catheter tip and bloodstream isolates were subjected to molecular identification by direct DNA sequencing of internally transcribed-spacer-(ITS-) 1 and ITS-2 regions of ribosomal DNA (rDNA). 

## 4. Molecular Identification of Yeast Isolates

The DNA from both cultured yeast isolates was prepared, the entire ITS region (containing the ITS-1, 5.8S rRNA and ITS-2) of rDNA was amplified by using panfungal primers ITS1 and ITS4, and both strands of amplified DNA were sequenced as described previously [10, 11]. The sequencing primers, in addition to the amplification primers, included ITS1FS, ITS2, ITS3, and ITS4RS [12]. The sequence of the entire ITS region was assembled and GenBank basic local alignment search tool (BLAST) searches (http://www.ncbi.nlm.nih.gov/BLAST/Blast.cgi?) were performed for species identification. 

An amplicon of ~550 bp was obtained for the ITS region from the catheter tip isolate Kw553-08 and BLAST search of the DNA sequence data showed none or only one nucleotide difference in the corresponding region sequences available in the databank from three reference strains, CBS 2606, CBS 5301, and CBS 2605 (GenBank accession nos. AY391845, AY391847 and AY391848, resp.) of *L. elongisporus*. Similarly, an amplicon of ~650 bp was obtained for the ITS region from the blood culture isolate Kw554-08,and BLAST search of the DNA sequence revealed 0, 0, and 1 nucleotide difference in the corresponding region sequences available in the databank from *Sterigmatomyces elviae* reference strains CBS 5922, CBS 7053, and CBS 7288, respectively (GenBank accession nos. AF444551, AF444512, and AF444517, resp.). Based on the previous observations that fungal strains belonging to same species exhibit >99% nucleotide identity in the ITS-1 and ITS-2 of the rDNA region [13–15], the molecular identity of our isolates Kw553-08 and Kw554-08 are described as *L. elongisporus *and* S. elviae*, respectively [16]. The DNA sequence data of the ITS region of our isolates have been deposited in the EMBL under the accession nos. FM253093 and FM253094. Isolates Kw553-08 and Kw554-08 have been deposited at Centraalbureau voor Schimmelcultures under accession no. CBS 10974 and CBS 11701, respectively.

## 5. Antifungal Susceptibility Testing

*In vitro* susceptibility of both isolates was determined by Etest (AB Biodisk, Solna, Sweden) on RPMI medium supplemented with 2% glucose as recommended by Clinical Laboratory Standards Institute [17]. The test was performed according to the manufacturer's instructions and as described previously [18]. The antifungal strips used included amphotericin B, posaconazole, voriconazole, fluconazole, 5-flucytosine, and caspofungin. As per the Clinical Laboratory Standards Institute's minimum inhibitory concentration (MIC) breakpoints, *L. elongisporus *isolate Kw553-08 was considered susceptible to fluconazole (0.32 *μ*g/mL), voriconazole (0.002 *μ*g/mL), posaconazole (0.023 *μ*g/mL), caspofungin (0.094 *μ*g/mL), and 5-flucytosine (0.094 *μ*g/mL). On the other hand, the *S. elviae* isolate Kw554-08 was susceptible to fluconazole (6 *μ*g/mL), voriconazole (0.047 *μ*g/mL), and posaconazole (0.19 *μ*g/mL); however, it showed reduced susceptibility to caspofungin (>32 *μ*g/mL) and 5-flucytosine (>32 *μ*g/mL) and thus was considered resistant. 

## 6. Pathogenicity Test of *S. Elviae* Isolate in BALB/c Mice

Since* S. elviae *has never been reported as systemic human pathogen, its pathogenicity was determined in BALB/c mice. Ten normal and 10 immunosuppressed mice were injected intravenously with 1 × 10^7^ cells of freshly prepared suspension of* S. elviae *Kw554-08 in normal saline. The mice were immunosuppressed with three intramuscular injections of cortisone acetate (2 mg/mouse) given on the day of infection, on day 2 and day 4 after-infection [19]. None of the mice died during the two weeks followup and none of the organs showed gross lesions on necropsy examination. Hence, the isolate was considered as nonpathogenic for BALB/c mice.

## 7. Discussion

The present paper is noteworthy in that it describes the isolation of *L. elongisporus*, an ascomycetous yeast species, from a suspected fungemia case from the Middle East. Although the etiologic significance of isolation of *L. elongisporus *from the catheter tip culture of our patient was not accompanied by concomitant recovery from blood, its role as bloodstream pathogen capable of causing deep-seated infection has already been recognized [7–9]. The inability to culture the organism from blood is in conformity with previous observations showing that the blood culture positivity for *Candida* species even in autopsy-proven cases of invasive candidiasis remains low (~50%) [20]. The patient's favorable response to antifungal therapy is also consistent with the possible etiological role of *L. elongisporus* in fungemia since the isolate Kw553-08 recovered from the catheter tip was highly susceptible to fluconazole. Our paper also reinforces the previous observations that currently available phenotypic identification methods based on Vitek 2 or API 20C carbon assimilation profiles, misidentify isolates of *L. elongisporus* as *C. parapsilosis *[7]. It is probable, therefore, that *L. elongisporus* isolates may be occurring with greater frequency than hitherto recognized due to limitations of currently available phenotypic identification methods. Recent application of molecular methods have resulted in species-specific identification of closely related yeast species including *L. elongisporus* [21]. Application of molecular methods in one study involving 542 clinical isolates of *C. parapsilosis* obtained from 25 countries and previously identified by conventional phenotypic methods identified 10 isolates as *L. elongisporus*. Furthermore, 8 of 10 *L. elongisporus* originated from a single hospital in Mexico while one isolate each came from China and Malaysia [7]. Interestingly, all 10 bloodstream isolates formed distinct turquoise color on CHROMagar Candida instead of pink/lavender color colonies typical of *C. parapsilosis* isolates, thus facilitating their initial discrimination and subsequent confirmation as a distinct species by DNA sequencing [7]. Another case of fungemia due to* L. elongisporus* was also described subsequently from Malaysia while a case of endocarditis was reported recently from Australia [8, 9]. 

It is pertinent to mention here that *C. parapsilosis* complex members are the second leading cause of bloodstream infections in the Middle East [22, 23]. These organisms have also emerged as a leading cause of candidemia in low birth weight neonates [24, 25]. It is probable that some of the *C. parapsilosis* isolates identified by phenotypic methods alone at other geographical locations could also represent *L. elongisporus *or two other closely related species (i.e., *C. orthopsilosis* and* C. metapsilosis* now comprising* C. parapsilosis*-complex [23, 26]. Since *C. parapsilosis* and *L. elongisporus* exhibit similar antifungal susceptibility profiles [7–9, 27], it may not be of immediate concern to clinicians while treating such patients as the cases of *C. parapsilosis* fungemia. However, so far, only a small number of clinical isolates of *L. elongisporus*,* C. orthopsilosis,* or *C. metapsilosis* have been tested. As more isolates become available, it is probable that clinically significant differences in MICs against some antifungal agents may be observed necessitating species-specific identification in clinical laboratories.

The clinical significance of *S. elviae* in our patient remained equivocal. Although the first two strains of *S. elviae* were isolated from eczematous cutaneous lesions of two Finnish patients in 1966 and 1968 [28, 29], their role as a systemic human pathogen has never been established. Although our isolate as well as the initial two strains of *S. elviae* were able to grow up to 40°C, however, the organism was not found pathogenic for BALB/c mice [29]. Our findings also showed that *S. elviae* isolate Kw554-08 was not pathogenic for immunosuppressed mice. Based on these observations, we considered *S. elviae* as a contaminant probably originating from improper skin decontamination during collection of one blood sample. The inability to culture *L. elongisporus* from blood samples may be attributed to the transient nature of fungemia, intermittent seeding of the yeast from the catheter, and its rapid clearance following fluconazole therapy. The low blood culture positivity for *Candida* spp. commonly seen even in proven cases of candidemia could be another possibility. 

## 8. Conclusions

The isolation of *L. elongisporus*, a recently recognized bloodstream pathogen, has been reported from the Middle East. The identity of the isolate was confirmed by ribosomal DNA sequencing. This paper underscores the importance of molecular methods in achieving correct identification of yeast species that may remain unidentified by conventional methods.
